# Supplementary material for: Planar thermal Hall effect from phonons in a Kitaev candidate material
Source: Nat Commun. 2024 Apr 25;15:3513. doi: 10.1038/s41467-024-47858-5 (PMC11045815; doi:10.1038/s41467-024-47858-5)
Supplement: Supplementary file 1 — supplementary information [file 41467_2024_47858_MOESM1_ESM.pdf]

Supplementary Information

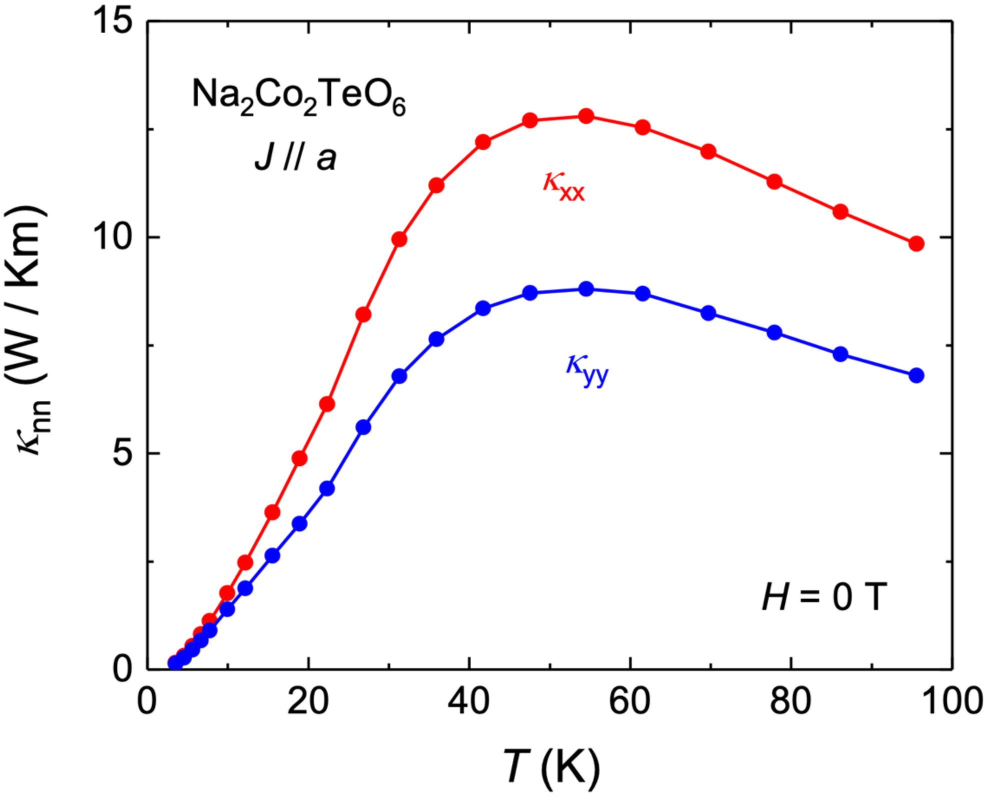

Supplementary Fig. 1 | Thermal conductivity data in $Na_2Co_2TeO_6$ with *J // a*.

Red curve is the thermal conductivity κ_{xx} vs temperature T in $Na_2Co_2TeO_6$ sample A measured at a magnetic field H = 0 T with heat current J // a. The thermal conductivity κ_{yy} at H = 0 T with J // a^* in that sample (blue curve) is estimated by multiplying the κ_{xx} data by the anisotropy factor κ_{yy}/κ_{xx} reported in Ref. [27]. a denotes the zigzag direction (perpendicular to the Co-Co bond), a^* denotes the armchair direction (parallel to the Co-Co bond).

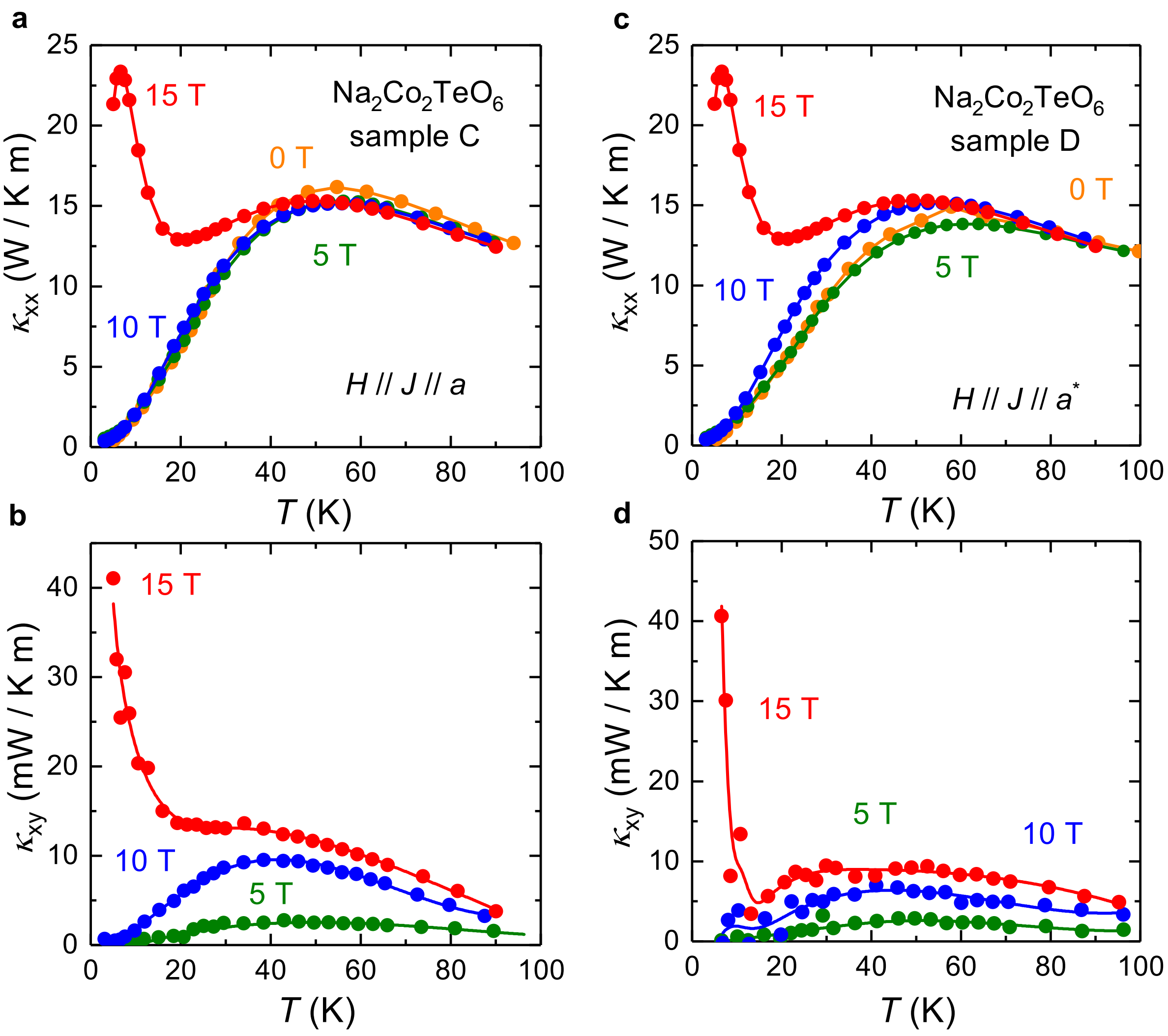

Supplementary Fig. 2 | Thermal transport in samples C and D.

Thermal conductivity κ_{xx} vs temperature T in $Na_2Co_2TeO_6$, measured on sample C **(a)**, measured with H // J // a, and sample D **(c)**, measured with H // J // a^*, at a magnetic field $H = 0$, 5, 10 and 15 T. Corresponding thermal Hall conductivity κ_{xy} for sample C **(b)** and sample D **(d)**. a denotes the zigzag direction (perpendicular to the Co-Co bond), a^* denotes the armchair direction (parallel to the Co-Co bond).

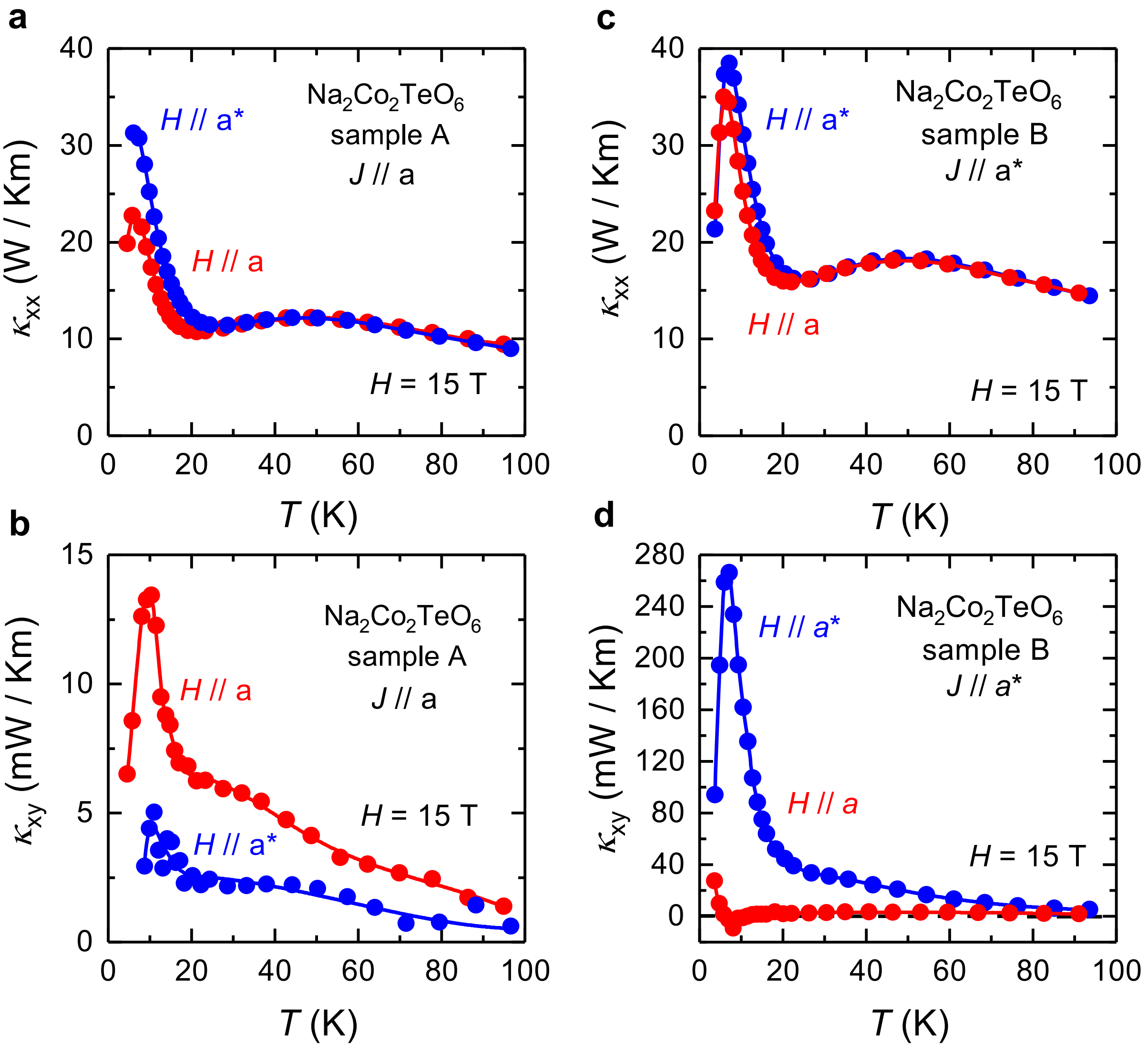

Supplementary Fig. 3 | Comparing *H // J* and *H* ⊥ *J* in samples A and B.

a) Thermal conductivity κ_{xx} vs temperature T and **b)** thermal Hall conductivity κ_{xy} vs T in $Na_2Co_2TeO_6$ measured on sample A with the heat current J // a for H // a (red) and H // a^* (blue), at a magnetic field H = 15 T. **c, d)** Corresponding data for sample B, measured with J // a^*. We observe that κ_{xy} measured in the configuration $H \perp J$ is much smaller than in the configuration H // J. a denotes the zigzag direction (perpendicular to the Co-Co bond), a^* denotes the armchair direction (parallel to the Co-Co bond).
